# Therapeutic challenges and current immunomodulatory strategies in targeting the immunosuppressive pancreatic tumor microenvironment

**DOI:** 10.1186/s13046-019-1153-8

**Published:** 2019-04-15

**Authors:** Chin-King Looi, Felicia Fei-Lei Chung, Chee-Onn Leong, Shew-Fung Wong, Rozita Rosli, Chun-Wai Mai

**Affiliations:** 1 School of Postgraduate Studies, International Medical University, Kuala Lumpur, Malaysia; 2 Mechanisms of Carcinogenesis Section (MCA), Epigenetics Group (EGE) International Agency for Research on Cancer, World Health Organization, Lyon, France; 3 School of Pharmacy, International Medical University, Kuala Lumpur, Malaysia; 4 Center for Cancer and Stem Cell Research, Institute for Research, Development and Innovation (IRDI), International Medical University, Kuala Lumpur, Malaysia; 5 School of Medicine, International Medical University, Kuala Lumpur, Malaysia; 6 UPM-MAKNA Cancer Research Laboratory, Institute of Bioscience, Universiti Putra Malaysia, Sri Kembangan, Selangor Malaysia

**Keywords:** Pancreatic cancer, Immunotherapy, Tumor microenvironment

## Abstract

**Background:**

Pancreatic cancer is one of the most lethal type of cancers, with an overall five-year survival rate of less than 5%. It is usually diagnosed at an advanced stage with limited therapeutic options. To date, no effective treatment options have demonstrated long-term benefits in advanced pancreatic cancer patients. Compared with other cancers, pancreatic cancer exhibits remarkable resistance to conventional therapy and possesses a highly immunosuppressive tumor microenvironment (TME).

**Main body:**

In this review, we summarized the evidence and unique properties of TME in pancreatic cancer that may contribute to its resistance towards immunotherapies as well as strategies to overcome those barriers. We reviewed the current strategies and future perspectives of combination therapies that (1) promote T cell priming through tumor associated antigen presentation; (2) inhibit tumor immunosuppressive environment; and (3) break-down the desmoplastic barrier which improves tumor infiltrating lymphocytes entry into the TME.

**Conclusions:**

It is imperative for clinicians and scientists to understand tumor immunology, identify novel biomarkers, and optimize the position of immunotherapy in therapeutic sequence, in order to improve pancreatic cancer clinical trial outcomes. Our collaborative efforts in targeting pancreatic TME will be the mainstay of achieving better clinical prognosis among pancreatic cancer patients. Ultimately, pancreatic cancer will be a treatable medical condition instead of a death sentence for a patient.

## Background

Pancreatic cancer is an aggressive malignancy usually diagnosed at an advanced stage with very limited therapeutic options. According to GLOBOCAN 2018, pancreatic cancer is the seventh leading cause of cancer death in both males and females [1]. The estimated 5-year survival rate for pancreatic cancer is less than 5%, which is the lowest among other cancers [2]. Pancreatic cancer is expected to become the second leading cause of cancer death by 2030 in the United States (US), surpassing breast, prostate and colorectal cancers [3]. One of the backbone chemotherapeutic agents that has been used since the late nineties for pancreatic cancer is gemcitabine [4]. However, clinical data have shown that a large number of patients do not respond to gemcitabine monotherapy, and thus it is believed that the tumor cells have acquired intrinsic or chemoresistance towards gemcitabine treatment [5]. Since then, combinational therapies such as FOLFIRINOX [6] and the combination of gemcitabine with albumin-bound paclitaxel (nab-paclitaxel) [7], have been shown to be an alternative strategy, with only a marginal increase in overall survival (OS) but patients would then suffer with increased toxicity compared to gemcitabine alone.

Recently, the application of immunotherapies to boost effector T cells to kill cancer cells has generated much excitement. Particularly, strategies targeting immune checkpoint molecules through inhibition of programmed death 1 (PD-1) and cytotoxic T lymphocyte antigen-4 (CTLA-4) have demonstrated clinical benefit in several malignancies, such as melanoma [8, 9], Hodgkin’s lymphoma [10], and non-small cell lung cancer (NSCLC) [11]. This has therefore raised hope for pancreatic cancer patients. However, clinical studies have shown that checkpoint inhibition therapy alone is insufficient in treating patients with pancreatic cancer [12, 13]. The tumor microenvironment (TME) of pancreatic cancer is unique and may promote tumor evasion as well as conferring resistance to therapeutic agents including the immune therapies [14]. Based on the literature, compounds, or therapeutic approaches that targeting cytochromes [15] or immune mediators such as legumain [16] and Toll-like receptors [17] may reduce the influence of the tumor microenvironment on tumor progression. Some studies also suggested that nanotechnology or micronized chemotherapy deliveries may enhance the clinical outcomes among cancer patients [18]. However, the evidence for the effectiveness such approaches in targeting pancreatic tumor microenvironment is not clearly defined due to the lack of in-depth studies. Therefore, more thorough clinical research concerning the pancreatic TME is greatly needed.

In this review, we will explore the unique TME of pancreatic cancer that may act to limit the treatment efficacy of immunotherapy. We critically discuss the available treatment strategies for this disease. We will summarize findings on recent and ongoing combination immunotherapies currently being evaluated in clinical trial settings that focused on improving the effectiveness of immunotherapy in pancreatic cancer.

## Main text

### Characteristics of TME in pancreatic cancer

**Fig. 1:**
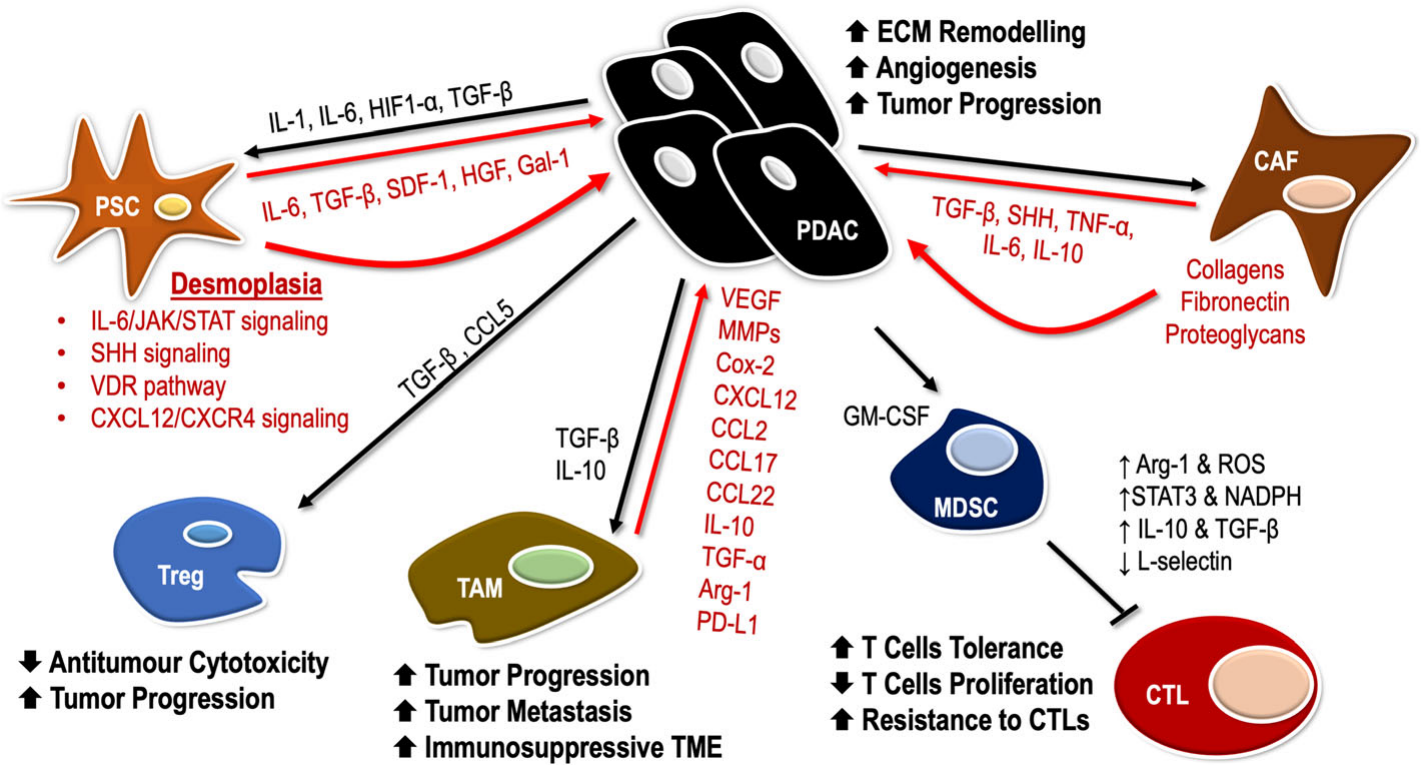
Crosstalk of pancreatic cancer cells with other cells in the tumor microenvironment

Pancreatic cancer features a highly immunosuppressive microenvironment, characterized by a dense desmoplastic stroma, which impedes blood flow to the area, inhibits drug delivery, and suppresses antitumor immune response [19]. This favors cancer progression by protecting pancreatic tumors from immune surveillance as well as regional and distant metastasis [20]. Additionally, the hypoxic environment, acidic extracellular pH, and high interstitial fluid pressure in the TME also act to enhance tumorigenesis and tumor progression [21]. In order to create an environment that is conducive for tumor growth, tumor supporting cells are upregulated, whereas the immune cells are downregulated in the TME of pancreatic cancer. Cells such as myeloid-derived suppressor cells (MDSCs), tumor-associated macrophages (TAMs), regulatory T cells (Tregs), fibroblasts, and mast cells are upregulated in the TME, ultimately protecting tumor cells from being eliminated by the immune system; on the other hand, natural killer (NK) cells and CD8^+^ T cells that act to destroy tumor cells are downregulated [22]. The interaction between the tumor cells and TME components acts to facilitate the development and progression of tumors, as well as invasion, and metastasis (Fig. 1) [23].

### Cancer-associated fibroblasts (CAFs)

CAFs are an important component within the tumor stroma. They develop from bone marrow-derived mesenchymal stem cells (MSCs), pancreatic stellate cells (PSCs), and resting fibroblasts in the pancreas through epithelial-mesenchymal transition (EMT). Activation of CAFs is induced by cancer-secreting cytokines such as TGF-β, sonic hedgehog (SHH), TNF-α, IL-6, and IL-10 [24]. CAFs have been shown to be associated with tumor progression by stimulating the production of growth factors, inflammatory cytokines and chemokines, pro-angiogenic factors, and metabolites that stimulate signaling pathways in cancer cells [25]. Additionally, CAFs are also involved in remodeling of the ECM to form a pro-cancerous microenvironment by producing excessive amounts of structural matrix components, such as collagen, fibronectin, and proteoglycans [26, 27]. This in turn promotes the aggressive biology of pancreatic cancer, resulting in tumor proliferation, angiogenesis, metastasis, survival, and resistance to therapy.

Additionally, the migration and proliferation of pancreatic cancer is also supported by CAFs expressing fibroblast activation proteins (FAP). Overexpression of FAP in tumor cells resulted in increased tumorigenicity and enhanced tumor progression [28, 29]; the enzymatic activity of FAP plays an essential role in FAP-driven tumor growth in a murine xenograft model, as evidenced by inhibition of FAP enzymatic activity being associated with tumor attenuation [30]. As expected, higher levels of FAP expression in patients were associated with shorter overall survival compared with patients showing lower FAP expression, suggesting that FAP is important for tumor progression and metastasis in pancreatic cancer [31].Deletion of FAP gene and pharmacologic inhibition of FAP enzymatic activity reduced the tumor burden probably through disturbing stromagenesis, angiogenesis, and promotes nti-tumor immune responses. [32–34]. Administration of FAP-CAR T cells into tumor-bearing mice significantly reduced tumor growth and induced apoptosis of tumor cells [31]. The degree of desmoplasia was found to be reduced in highly desmoplastic lung cancer xenografts, with a marked disruption of adenocarcinoma ductal-like structure of the tumor nodules, a decrease in collagen and fibronectin content, and an increase in necrosis in FAP-CAR T cell-treated tumors. This in turn promotes the recruitment of immune cells, consequently augmenting antitumor immunity [31]. Similar results were observed in murine models of pancreatic cancer, where FAP-CAR T cells significantly inhibited the growth of non-immunogenic tumor [31–34]. Depletion of FAP-expressing stromal cells resulted in a better immunological response and a lower tumor burden [29, 35],These findings may suggest therapeutics that selectively target FAP-expressing cells, but not other cancer-associated stromal cells, may result in a better prognosis.

### Pancreatic stellate cells (PSCs)

More than 80% of human pancreatic cancer tissues are associated with a highly desmoplastic stroma, and pancreatic stellate cells (PSCs) are creators of this stroma in pancreatic cancer [23]. In the non-inflamed pancreas, quiescent PSCs have a lower mitotic index [21] and are involve in maintaining tissue homeostasis [36]. In pancreatic ductal adenocarcinoma (PDAC), quiescent PSCs are activated by environmental stress (oxidative stress and hypoxia), cellular factors [IL-1, IL-6, hypoxia inducible factor 1-α (HIF1-α), and TGF-β], as well as molecular signaling pathway such as the PI3K pathway, and are transformed into myofibroblast-like cells [21, 37, 38]. These activated PSCs acquire proliferative capability and are associated with an upregulation of matrix metalloproteinases (MMPs) and extracellular matrix proteins (ECMs) [38], which enhance sustained fibrosis and tumor angiogenesis via the production of vascular endothelial growth factors (VEGFs) [21], creating a physical barrier to therapeutic agents [36]. Additionally, activated PSCs promote tumor progression by secreting IL-6, TGF-β, stromal cell-derived factor-1 (SDF-1), hepatocyte growth factor (HGF), and galectin-1 (Gal-1, 38]. Notably, PSCs play a key role in inducing desmoplastic reactions in the TME of pancreatic cancer. Recent evidence has demonstrated that PSCs can drive desmoplasia via several signaling pathway, such as IL-6/JAK/STAT signaling, paracrine Sonic Hedgehog (SHH) signaling, the vitamin D Receptor (VDR) pathway, and CXCL12/CXCR4 signaling axis. In addition, the secretion of CXCL12 by PSCs results in limiting the migration of CD8^+^ T cells to juxtatumoral stromal compartments, protecting the tumor cells from the cytotoxicity of CD8^+^ T cells [39]. Overexpression of Gal-1 in PSCs promotes immunosuppression by inducing apoptosis of CD4^+^ and CD8^+^ T cells and increasing the secretion of Th2 cytokines (IL-4 and IL-5), while reducing the secretion of Th1 cytokines (IL-2 and IFN-γ) [40].

### CD4^+^CD25^+^Foxp3^+^ regulatory T cells (Tregs)

CD4^+^CD25^+^Foxp3^+^ regulatory T cells (Tregs) also defined as suppressor T cells, play an important role in immunosuppression via expression of CTLA-4 and secretion of IL-10 and TGF-β [41]. In the physiological state, Tregs help to regulate immunological tolerance to self-antigens and prevent autoimmunity, whereas, in tumors, they suppress antitumor immune responses by inhibiting effector T cell functions [41]. The migration of circulating Tregs into the pancreatic cancer TME is controlled by the interactions between tumor chemokines and their ligands/receptors. It was also shown that in both human PDAC and a mouse pancreatic tumor model, the cancer cells produced higher level of ligands for chemokine receptor 5 (CCR5), while Tregs expressed CCR5. When CCR5/CCL5 interaction is diminished or blocked, migration of Tregs to tumor is reduced, and even the size of tumor became smaller [42]. In addition, TGF-β was found to be involved in the recruitment of Tregs in pancreatic cancer. Tregs secrete TGF-β to suppress antitumor cytotoxic activity; on the other hand, pancreatic cancer induces Tregs in the presence of TGF-β [43]. In a murine model of pancreatic cancer, the conversion of CD4^+^CD25^−^ naïve T cells into Foxp3^+^ Tregs was shown to be mediated by TGF-β [44]. In PDAC, poorer prognosis of patients is associated with a higher proportions of Tregs in tumor-infiltrating lymphocytes (TILs) [45]. The increased infiltration of Tregs into the TME is also positively correlated with tumor progression [42] and inversely correlated with the presence of CD8^+^ T cells [45]. In pre-clinical studies, the depletion of Tregs with anti-CTLA-4, anti-CD25 or CCR5 inhibitor r reduced tumor growth and prolonged the animals’ survival by enhancing the activation of tumor specific T cells. [41, 42, 46, 47].

### Myeloid-derived suppressor cells (MDSCs)

MDSCs are immature myeloid cells that suppress the immune response in pancreatic cancer. Granulocytic MDSCs express CD33, CD11b, and IL-4Rα, with low levels of CD15 and high levels of arginase. On the other hand, monocytic MDSCs express the same markers as granulocytic MDSCs, but with lower levels of CD15, and also express CD14 apart from arginase. They also express inducible nitric oxide synthase (iNOS) [48]. In pancreatic cancer, the proliferation and migration of MDSCs from bone marrow into the TME is consistently induced by granulocyte macrophage colony-stimulating factor (GM-CSF) [38]. In tumors, MDSCs suppress the antitumor activity of both CD8^+^ and CD4^+^ T cells, and expand immunosuppressive Tregs. Moreover, MDSCs can block innate immunity by converting M1 macrophages which promote tumor regression into M2 phenotypes which facilitate tumor progression [49] as well as suppressing NK cells antitumor cytotoxicity [50]. This repolarization is the result of cross-talk between MDSCs and macrophages, in which the production of IL-10 by MDSCs is increased, while increasing the production of IL-12 by macrophages [49]. MDSCs can suppress T cell activity via multiple mechanisms; these mechanisms include the depletion of arginine (Arg) [51], the secretion of reactive oxygen species (ROS) [52] and downregulation of L-selectin [53]. In tumors, MDSCs synthesize high levels of arginase-1 (Arg-1) to deplete Arg in the TME [51]. Depletion of Arg results in decreased expression of the CD3ζ chain, leading to the reduction of IL-2 and IFN-γ, and, consequently, inhibition of the proliferation of T cells and induction of T cell tolerance [23]. The secretion of cytokines such as IL-10 and TGF-β [38] and increased activation of STAT 3 and NADPH [54] can induce MDSCs to release ROS, resulting in oxidative stress in T cells [38] as well as the suppression of CD8^+^ T cell responses [52]. The production of free radical peroxynitrite (PNT) was shown to stimulate MDSCs to mediate tumor cells resistance to CTLs, through T cell tolerance and nitration of T cell receptors (TCRs) on the T cell surface. This causes TCRs to lose the ability to recognize and bind to specific peptide/MHC complexes and perform subsequent antitumor activity. Another mechanism employed by MDSCs to inhibit antitumor immunity is by impairing the homing of T cells to lymph nodes via the downregulation of L-selectin. Adaptive T cell-mediated antitumor immunity requires the activation of antigen-naïve T cells; L-selectin is important in facilitating the extravasation of leucocytes to lymph nodes where they become activated prior to being directed to inflammatory sites such as the TME [53, 54]. By downregulating L-selectin expression in CD8^+^ and CD4^+^ T cells, MDSCs impair the T cell trafficking pattern, thereby inhibiting the activation of T cells [54].

### Tumor associated macrophages (TAMs)

Macrophages in tumors are usually defined as TAMs and often express the M2 phenotype. In general, M1 macrophages facilitate tumor regression and Th1 responses by secreting tumor necrosis factor-α (TNF-α) and IL-12; on the other hand, M2 macrophages display an immune suppressive phenotype and release IL-10 which promotes a Th2 response [55]. In human PDAC, macrophages are prominent compared with the healthy pancreas. Macrophages were found to infiltrate in low grade, pre-invasive pancreatic tumor lesions and persist in invasive pancreatic cancer in a mouse model [56]. The percentages of MDSCs and TAMs are elevated significantly with the progression of pancreatic cancer; conversely, the percentages of CD8^+^ and CD4^+^ T cells are significantly reduced [23]. As a result, macrophages play a critical role in facilitating tumor progression, angiogenesis, stromal remodeling, and metastasis in pancreatic cancer [57]. TAMs can facilitate tumor metastasis by secreting matrix proteins and proteases such as serine proteases, matrix metalloproteinases (MMPs), and cathepsins which act to modify the extracellular matrix (ECM) composition [58]. The overexpression of MMP9 induced via the interaction of macrophage inflammatory protein-3 alpha (MIP-3α) with its receptor, increases the expression of CCR6 on pancreatic cancer cells, consequently increasing the invasion of pancreatic cancer cells [59]. Macrophages also drive the development of an immunosuppressive environment by secreting angiogenic factors such as thymidine phosphorylase (TP), vascular endothelial growth factor (VEGF), MMPs, cyclooxygenase-2 (Cox-2), CXCL12, and CCL2, as well as immunosuppressive factors such as IL-10, TGF-α, Arg-1, CCL17 and CCL22 [58]. TAMs also promote apoptosis of T cells by expressing programmed death-ligand 1 (PD-L1) on their cell surface [58]. As a result, TAMs may contribute to the pancreatic tumor immune evasion, resulting in the survival of these tumors, despite aggressive chemotherapy .

### Potential factors that limit the efficacy of immunotherapy

Pancreatic cancers have an intrinsically low mutational burden, and thereby exhibit low levels of neoantigen-expression. As the mutational load and neoantigen burden are positively correlated with the efficacy of immunotherapy [60, 61], cancers with higher mutational loads, which generate more neoantigens, could elicit enhanced T cell recognition. In contrast, cancers with a low mutational load, for instance pancreatic cancer, only occasionally produce neoantigens, as their average mutation rate accounts only for one mutation per megabase (Mb), compared to 11 mutations per Mb for melanomas [62]. Tumor immunogenicity is the key initial step in launching effective antitumor responses to immune checkpoint blockade. As a result of the lack of sufficient neoantigen targets, the lower level of TILs in the TME creates a non-immunogenic or ‘cold’ microenvironment, thereby limiting effective T cell responses and impeding the efficacy of immunotherapy [63].

Other mechanisms of resistance to immune checkpoint blockade in pancreatic cancer include aberrant expression of immune checkpoints such as PD-L1 on the tumor cell surface, downregulation of antigen presenting MHC molecules, reduced Fas receptor signaling and therefore a reduction in counterattack by T cells via the expression of Fas ligands [64, 65]. In addition, the establishment of a highly desmoplastic TME by stromal cells creates a therapeutic barrier in treating pancreatic cancer [66]. For instance, it is especially difficult to deliver drugs to pancreatic tumors compared with other solid tumors due to their hypovascular and poorly perfused nature [67]. The presence of stromal components in pancreatic cancer increases the interstitial fluid pressure, consequently inhibits the drug from penetrating the interstitial tissue [66, 67]. Furthermore, the formation and function of the blood vasculature can be inhibited by fibroblasts and the fibrotic stroma in pancreatic cancer [67], thereby diminishing drug delivery via the blood and reducing the effectiveness of chemotherapy.

### Strategies for Cancer immunotherapy

In recent years, cancer immunotherapy is gaining much attention in view of its promising efficacy. One cancer immunotherapy comprises antibodies that target immune checkpoints. Ipilimumab, the first anti-CTLA-4 antibody was approved by the US Food and Drug Administration (FDA) in 2011 for melanoma [60]. Compared with placebo, ipilimumab significantly improved overall survival, this being, respectively, 9.1 and 11.2 months in patients with previously untreated metastatic melanoma [68]. PD-1 inhibitors such as nivolumab and pembrolizumab have been approved for melanoma treatment [8, 9] and are still being tested in pancreatic cancer clinical trials [12, 69]. Overall survival of melanoma patients who received nivolumab was considerably longer than progression-free survival, with an acceptable long-term safety profile [9]. Similarly, pembrolizumab also showed promising results in decreasing tumor size in melanoma patients [70, 71]. Another three anti-PD-L1 antibodies, atezolizumab, durvalumab and avelumab, have also been approved by the FDA [72]. The safety profile and clinical activity of atezolizumab were studied in renal cell carcinoma (RCC) where the drug showed promising antitumor activity in patients with metastatic disease. About 46% of patients with clear cell RCC had tumor shrinkage with an overall survival of 23.9 months [73]. Durvalumab was approved by the FDA in 2018 to treat patients with unresectable, stage III NSCLC. Patients receiving durvalumab demonstrated significant improvement in progression-free survival (16.8 months) compared with patients who received placebo, 16.8 months (5.6 months) [74]. Avelumab received accelerated approval by the FDA for the treatment of metastatic Merkel cell carcinoma (MCC), a rare type of skin cancer in 2017 [75]. The binding of avelumab to PD-L1 can inhibit the interaction of PD-L1 with PD-1, consequently restoring the immune response as well as antitumor activity [75]. The overall response rate (ORR) was 33%, while the estimated one-year overall and progression-free survival were 52 and 30%, respectively [75].

Durable clinical responses and prolonged survival rate have been shown in patients with melanoma and highly immunogenic cancers using monoclonal antibodies (mAb) targeting CTLA-4 or PD-1 [68]. However, based on the early clinical trials, checkpoint inhibitors, such as anti-CTLA-4, anti-PD-1 or anti-PD-L1, are ineffective when used as monotherapy in the treatment of pancreatic cancer [76]. This inefficacy is likely due to the low immunogenicity and non-inflamed phenotype (low levels of TILs) of pancreatic cancer as mentioned previously [61]. No objective responses were observed in advanced and metastatic pancreatic cancer patients treated with ipilimumab, indicating that ipilimumab alone is not an effective therapy for advanced pancreatic cancer [77]. Similarly, in a phase I study with anti-PD-L1 mAb alone, a 0% overall response rate (ORR) was observed in advanced pancreatic cancer patients [12]. Therefore, immunotherapy is not always effective and requires further development along with new combination strategies in order to enhance its efficacy. These combination therapies can be classified based on their strategic targets: firstly, to promote T cell priming by enhancing TAA (tumor associated antigen) presentation; secondly, to target the immunosuppressive environment thus relieving immunosuppression; and thirdly, to bring more TILs into the TME by breaking down the desmoplastic barrier [60].

### Enhancing T cell priming

Insufficient T cell priming is a root cause of ‘cold’ tumors and immune checkpoint unresponsiveness [78]. Antigen presenting cells (APCs), particularly dendritic cells (DCs) are essential for T cell priming which generates effective antitumor T cell responses. Of note, higher levels of circulating DCs have been associated with better survival rate in pancreatic cancer patients [79]. Therefore, the TME would first need to be primed with effector T cells before immune checkpoint inhibitors could play their roles. Combining techniques that inhibit immunosuppressive signaling in TME while activating tumor-specific T cells against tumor cells seems to represent the most promising approach for immunotherapy in the treatment of pancreatic cancer.

1. Combination of immune checkpoint therapy with chemotherapy

Chemotherapy has been recognized as one important treatment strategy in human malignancy. However, the use of chemotherapy along with other clinically use agents may achieve better clinical outcomes. [80] The recruitment and activation of DCs [81] as well as the induction of the release of tumor specific antigens [82] may have a critical role in achieving this synergism. DCs are critical for T cell priming and the activation of a specific CD8^+^ T cell immune response. Tumor antigens must be presented by APCs such as DCs to naïve CD8^+^ T cells via cross-presentation. However, tumor-infiltrating DCs may be functionally impaired or may display defective migration into tumor-draining lymph nodes [81]. This failure can be reversed by using a chemotherapeutic agent to induce the recruitment of DCs to the tumor sites, thereby further enhancing the cross-presenting potential of tumor infiltrating DCs, which is crucial for the subsequent tumor antigen-specific cellular priming [83]. Unfortunately, both acute and cumulative toxicities to normal tissues caused by the delivery of cytotoxic agents have limited the dose and duration of treatment [84]. Therefore, the combination of chemotherapy and immunotherapy could potentially enhance the effectiveness of cancer treatment through different mechanisms of actions.

A recent phase I study (85) evaluated the safety profile of the combination of gemcitabine with an anti-CTLA-4 mAb (tremelimumab; CP-675,206) in metastatic pancreatic cancer patients. Tremelimumab is a fully humanized mAb that antagonizes the binding of CTLA-4 to B7–1 as well as B7–2, blocking the co-inhibition signal, thus leading to T cell activation. This combination therapy resulted in tolerable side effects, with a median overall survival of 5.3, 8.0, and 7.5 months for patients who received 6, 10 and 15 mg/kg of tremelimumab, respectively [85]. Among 28 patients, seven patients showed stable disease for more than 10 weeks; two patients who received 15 mg/kg tremelimumab managed to achieve a partial response at 8 weeks. Guo and coworkers also highlighted preliminary results from an ongoing phase Ib study of ipilimumab and gemcitabine on unresectable pancreatic cancer patients; this similarly showed a partial response and stable disease. Immunohistochemistry analysis further showed that the positive expression of PD-L1 was correlated with a worse overall survival [60].

In a murine model of pancreatic cancer, treatment with anti-PD-L1 or anti-PD-1 mAbs enhanced the infiltration of CD8^+^ T cells and significantly increased the expression of IFN-γ, granzyme B and perforin in implanted tumors. The blockade of PD-L1 promoted infiltration of CD8^+^ T cells into the tumor site and induced local immune activation. Furthermore, the combination of gemcitabine with anti-PD-L1 mAb exhibited significant synergistic effect, eliciting a complete response without overt toxicity in treated mice [86]. A clinical study (NCT01313416) on the combination of gemcitabine and pidilizumab (CT-011), a humanized mAb against PD-1 has been closed to enrolment. CT-011 is designed to specifically bind to PD-1, thus inhibiting PD-1 activity and attenuating apoptotic processes of effector or memory T lymphocytes, ultimately resulting in the activation of a CTL antitumor immune response [69]. CT-011 has been studied in murine models of other cancers, including leukemia, melanoma, lung cancer and colorectal carcinoma. CT-011 treatment resulted in reduced tumor growth and prolonged survival in tumor bearing nude mice. CT-011 also provided protection against tumor re-challenge.

(2) Combination of immune checkpoint therapy with cancer vaccines

Cancer vaccines are designed to augment antigen presentation and activate effector T cells. When vaccines containing target tumor antigens are given, host APCs would present these antigens to effector T cells which are then primed to kill tumor cells expressing these specific antigens, ultimately stimulating the development of antitumor immunity. One of the most extensively studied cancer vaccines is GVAX. It is made up of allogenic irradiated pancreatic cancer cells that have been genetically engineered to produce GM-CSF, a cytokine that further stimulates antigen presentation, T cell priming, and promotes cytolytic activity against tumor cells [60, 87]. In a phase II adjuvant study, GVAX induced the expansion of pancreatic cancer-specific CD8^+^ T cells and consequently improved the overall survival of patients. Patients who remained disease free after combination therapy (chemotherapy, radiotherapy and/or immunotherapy) generated lymphocytes that could respond to a greater variety of tumor associated antigens, suggesting that immunotherapy can be used either as an adjuvant treatment or in combination with other conventional therapies [88]. Immunohistochemical analysis (IHC) revealed the formation of intratumoral tertiary lymphoid aggregates in 33 out of 39 GVAX vaccinated pancreatic cancer patients; these aggregates were not observed in tumors of non--vaccinated patients [89]. The aggregates developed in response to antigen exposure and are composed of APCs and B cells, as well as naïve and activated T cells. These aggregates also indicated that vaccine-based immunotherapy plays a role in inducing an adaptive immune response in the TME in which GVAX could alter the pancreatic cancer TME, thus facilitating the infiltration of functional immune effector cells, and cconverting pancreatic cancer from non-immunogenic into immunogenic neoplasms [89].

Conceivably, the combination of an immune checkpoint inhibitor with vaccine therapy may synergistically induce an antitumor immune response. A preclinical study in melanoma indicated that a combination of GVAX with immune checkpoint blockade effectively eradicated tumors in mice which suffered from B16-BL6, an induced, highly non-immunogenic melanoma, that is resistant to immune checkpoint blockade therapy alone [90]. In a randomized phase Ib study, patients receiving the combination of GVAX and anti-CTLA-4 mAb (ipilimumab) had improved overall survival (median 5.7 months) compared to patients receiving ipilimumab alone (3.6 months) Patients with prolonged survival showed a higher number of tumor-infiltrating CD8^+^ T cells in the TME, indicating an improved antitumor immune response. Compared to ipilimumab alone, the percentage of patients surviving after 1 year was higher (27%) in the combination therapy arm versus 1% [91].These data suggested that T cells first need to be primed for their activation by T cell modulating agents such as ipilimumab.

A similar treatment was tested in preclinical murine models of pancreatic cancer using GVAX plus anti-PD-1 therapy. Combination therapy was found to significantly improve overall survival compared to PD-1 monotherapy. The secretion of interferon (IFN)-γ and the circulation of CD8^+^ T cells were increased in the TME of mice that received combination therapy with GVAX and PD-1 antibody blockade, compared with PD-1 monotherapy or GVAX therapy alone, indicating that the combination therapy could induce a synergistic effect antitumor immunity [92]. Furthermore, the addition of GVAX and low dose of cyclophosphamide to PD-1 blockade could downregulate the expression of CTLA-4 on T cells [92]. It is also important to highlight that an earlier study showed that PD-L1 was weakly expressed in both human and murine PDACs; the administration of GVAX significantly increased the expression of PD-L1 [92]. GVAX could increase the production of IFN- by infiltrating effector T cells, which may induce the upregulation of immunosuppressive mechanisms such as the overexpression of PD-L1 [89]. The upregulated expression of PD-L1 in tumor cells is associated with increased infiltration of immune cells and the formation of lymphoid aggregates, as well as an enhancement of the response rate of anti-PD-1 and anti-PD-L1 [89]. Lutz et al. also demonstrated an elevated expression of PD-L1 by monocytes and macrophages in the lymphoid aggregates that formed after GVAX therapy. In contrast, pancreatic tumors from non-vaccinated patients were rarely associated with PD-L1 expressing cells, indicating that PD-L1 expression is induced by vaccine treatment. Vaccine-primed patients would be better candidates than non-vaccinated patients for immune checkpoint therapy [89]. Therefore, the ineffectiveness of PD-L1 or PD-1 blockade in pancreatic cancer and the inability of PDAC to respond to a single checkpoint inhibitor therapy could be due to the lack of PD-1/PD-L1 expression and decreased infiltration of immune effector T cells to the tumor site. Thus, vaccine-based immunotherapy may overcome the resistance of pancreatic cancer towards immune checkpoint inhibitors by facilitating infiltration of tumor specific effector cells into the tumor site and upregulating PD-L1 expression, while immune checkpoint inhibitors may enhance the efficacy of vaccine-induced antitumor immune response by targeting PD-L1 signals on tumor cells [93]. To prove this concept, multiple clinical trials of PD-1/PD-L1 blockade in combination with GVAX vaccine therapy are ongoing (NCT02243371; NCT02648282; NCT02451982). For example, a randomized phase I/II clinical trial (NCT02451982) is ongoing to evaluate the efficacy of GVAX with or without anti-PD-1 mAb (nivolumab) as neoadjuvant or adjuvant treatment in resectable pancreatic cancer patients at Johns Hopkins University.

However, there are some limitations and several challenges have been associated with cancer immunotherapy targeting neoantigens. Antigens used in cancer vaccines should preferably be molecules that are different from normal cells, to ensure that antitumor immune response generated by vaccination only targeted on antigen bearing-tumor cells but not normal cells. Most tumor antigens are derived from mutated or modified self-proteins, leading to a risk of immune tolerance. This creates challenges in designing an appropriate cancer vaccine with reduced immune tolerance while eliciting antitumor immunity [94]. Another major concern is the heterogeneity of tumors. Neoantigens may be expressed in some, but not all, tumor cells in an individual patient, resulting in certain tumor cells escaping from immune surveillance [95]. The effectiveness of an allogeneic vaccine is highly correlated to the number of common tumor-associated antigens expressed by both the cancer and the allogeneic cell line. Therefore, the lack of strong immunogenicity of tumor neoantigens may greatly decrease the efficiency of vaccines [96]. In addition, since tumors frequently express antigens that are not specific to the tumor itself, treatment could ultimately lead to an increased risk of autoimmune-related adverse events, host immune suppression, and T cell exhaustion. Cancer vaccines are designed to target tumor neoantigens; tumor cells can evade destruction via developing antigen loss variants and this could increase the risk of autoimmunity. Therefore, to be recognized as an ideal cancer vaccine candidate, it should elicit strong immune response against the target cells, with antigen expression being restricted within the tumor itself, with minimal expression on normal tissues [97]. The highly immunosuppressive microenvironment of pancreatic cancer also contributes significantly to the unresponsiveness [98]. Furthermore, the use of allogenic therapies may promote tumor escape and drive further mutation. It has been hypothesized that the antigenic characteristics of allogeneic tumor cell vaccines developed from established cell lines might not be entirely the same as those of the tumor [99].

Accumulating evidence suggests that tumor neoantigens are one of the important targets for an antitumor immune response. Indeed, a higher neoantigen load and increased level of TILs are associated with improved survival in patients with colorectal [100] and endometrial cancer [101]. Therefore, the development of neoantigen cancer vaccines is highly dependent on the correct prediction and identification of neoantigens. Neoantigen prediction involves a series of computational steps, starting with the identification of mutations at the DNA level by comparing the whole exome sequences with those matched normal cells, followed by identifying targeted neoepitopes with the help of tumor RNA expression profiling, and finally determining the binding affinity of predicted epitopes to the MHC molecules with the use of software programs such as NetMHC or SYFPEITHI [98, 102]. However, there is the possibility of generating false positives (non-existent epitopes) or false negatives (missed epitopes). Computation of mutant allele coverage at the base level also adds difficulty in choosing the right transcript isoform to translate [102].

Reverse immunology has been postulated to be an efficient, high throughput approach for the discovery of tumor antigens. This approach involves the selection of peptides with strong binding to MHC molecules, such as proteins encoded by mutated oncogenes or genes that are highly expressed by tumors [103, 104]. This is predicted by in silico analysis using affinity prediction algorithms such as BIMAS and SYFPEITHY [103]. The most efficient binders are selected and bound onto APCs, and then used to activate CD8^+^ T cells that specifically recognize peptide-bound target cells [104]. High-throughput serological analysis of recombinant cDNA expression libraries (SEREX) has also been developed and widely used to identify and characterize the tumor antigens [105] in various types of cancers, including breast cancer [106], hepatocellular carcinoma [107], and gastric cancer [108], as well as pancreatic cancer [109]. The interaction of tumor antigens with antibodies in the autologous and allogeneic sera of cancer patients allows the identification of respective tumor antigens in the recombinant cDNA library [105]. The application of SEREX in identifying tumor antigens has also helped to define factors involved in tumorigenesis and further identify targets for diagnosis and vaccine-based therapy [107]. It allows a rapid identification of multiple tumor antigens and does not require the generation of tumor cell lines and pre-established CTL clones [110]. Furthermore, a proteome-based approach has recently been implemented in the prediction and identification of tumor neoantigens in cancer patients; this allows the screening of large number of patient sera and autoantigens [98]. In the future, the therapeutic efficacy of cancer vaccines can be enhanced by developing customized treatments based on the genomic and transcriptomic features of each patient, administered in conjunction with anti-immunosuppressive agents. Future directions also include strategies to increase the accuracy of choosing the right neoepitopes for personalized cancer vaccines and to overcome the occurrence of immune tolerance [96]. A continued focus on scientifically driven clinical trials is required to develop more potent and specific vaccines for the treatment of pancreatic cancer.

(3) Immune checkpoint therapy with agents that enhance T cell immunity

CD40 is a member of the TNF receptor family and is constitutively expressed on APCs. The binding of CD40 with its ligand (CD154), which is expressed on activated T cells, results in APCs activation, leading to activation of adaptive immunity. Ligation of CD40 on DC can increase the expression of MHC and co-stimulatory molecules, the production of pro-inflammatory cytokines, and enhanced T cell immunity [111]. In another preclinical trial, it was reported that CD40 activation itself was insufficient to induce a productive antitumor immune response, and required macrophages to rapidly infiltrate the tumor lesions, become tumoricidal and facilitate stroma depletion [112]. However, the use of a CD40 agonist was shown to promote maturation of macrophages and DCs, as well as cross-presentation of tumor antigens to CD8^+^ T cells, and to facilitate macrophage tumoricidal activity [113]. The administration of an agonist CD40 Ab with gemcitabine and nab-paclitaxel to mice resulted in macrophage-independent T cell immunity. This demonstrated that while the combination of gemcitabine and albumin- bound paclitaxel did not induce regression in established tumors, the use of an agonist CD40 Ab together with the chemotherapeutic agents significantly reduced tumor growth and improved survival compared to those receiving chemotherapeutic agents only.

Furthermore, the combination of CD40 mAb with chemotherapy was shown to enhance the efficacy of immune checkpoint therapy by priming the T cell response; treatment of tumor-bearing mice with this combination therapy resulted in reduced tumor progression and prolonged survival [63].CD40 mAb/chemotherapy was found to transform the TME of pancreatic cancer, resulting in reduced level of Tregs and increased infiltration of CD8^+^ T cells to the tumor site; this could further enhance the induction of a T cell response, and consequently augment the antitumor effects of anti-PD-1 in PDAC [63]. The induction of T cell immunity could transform pancreatic tumors that are completely resistant to immune checkpoint inhibitors into those in which tumor growth can be controlled with immune checkpoint blockade [63]. Luheshi and coworkers also demonstrated that the combination of an agonist CD40 mAb with PD-L1 blockade significantly delayed tumor growth and increased the overall survival in a murine model. All these data showed that CD40 mAbs play a role in stromal remodeling which transforms the immunosuppressive TME of pancreatic cancer, increases the infiltration of functional CD8^+^ T cells, enhances the expression of IL-2 and Th1 chemokines, and upregulates both the tumor and systemic PD-L1 expression; this could help to improve the sensitivity towards immune checkpoint therapy. In summary, while immune checkpoint monotherapy alone has minimal effects against PDAC, the combination of a CD40 mAb and PD-L1 blockade can improve the overall survival in comparison to either therapy alone.

Currently, a phase Ib clinical trial (NCT02304393) is assessing the safety, pharmacokinetics, pharmacodynamics and activity of a combination treatment of a CD40 mAb (RO7009789)) in conjunction with atezolizumab (anti-PD-L1) in patients with metastatic or locally advanced solid tumors. Another ongoing phase Ib/II study (NCT03214250) aims to investigate the safety and efficacy of a CD40 mAb (APX005M) administered with gemcitabine and nab-paclitaxel with or withoutanti-PD-1 mAb (nivolumab) in metastatic pancreatic cancer patients.

(4) Immune checkpoint therapy with adoptive T cell transfer

**Fig. 2:**
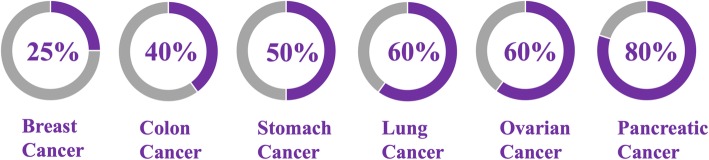
Frequency of mesothelin protein expression in various common solid malignancies

Other than CD40 mAbs, adoptive T cell transfer (ACT) also represents a promising immunotherapy approach for cancer. ACT uses genetically modified T cells to express chimeric antigen receptors (CAR), and has shown impressive activity in treating acute lymphoblastic leukemia [114, 115]. CARs are artificial receptors that are engineered to target specific antigens that are expressed in tumors but are not expressed, or expressed only at low levels, in normal tissues. By combining the antigen binding properties of mAb with lytic capacity of T cells, ‘off target’ effects and unspecific cytotoxicity can be minimized. CAR-T cells can also target cells in an MHC-independent fashion, bypassing tumor cell resistance towards MHC-restricted T-cell recognition [116, 117]. The efficacy of CAR T cells was found to be limited in solid tumors, particularly in pancreatic cancer, as there is a lack of an ideal target analogous to CD19 (B cell activation receptor), as well as the immunosuppressive environment of pancreatic cancer [118]. As a result, to treat pancreatic cancer, CAR is engineered to recognize mesothelin (membrane protein antigen) which is overexpressed in pancreatic cancer and other common solid tumors (Fig. 2) but is not expressed on T cells [118, 119] Mesothelin is an attractive target for immunotherapy due to its limited expression in normal tissues, its overexpression in malignant tissues, and its high immunogenicity [120]. Mesothelin might not be essential for the growth and reproduction of both wild type and mesothelin knockdown mice [121], although its aberrant or overexpression in preclinical and clinical studies showed that it plays an active role in both tumor malignancy and aggressiveness by promoting tumor proliferation, leading to invasion, metastasis, and conferring resistance towards cytotoxic agents [119].

In one of the completed phase I clinical trials (NCT01897415), T cells were engineered to express anti-mesothelin CAR and administered into patients with chemotherapy refractory metastatic pancreatic cancer. Of the six patients treated, two patients achieved stable disease in that study. Currently, an ongoing non-randomized phase I/II clinical trial (NCT01583686) is being conducted to determine the safety and efficacy of administering the engineered tumor fighting cells (anti-mesothelin cells) in metastatic cancer patients (ovarian, lung, cervical, mesothelioma and pancreatic cancer), followed by the administration of a preparative regimen (fludarabine, cyclophosphamide and aldeslekin). Furthermore, an ongoing non-randomized phase I study (NCT03323944) is evaluating the safety and feasibility of transducing meso-cells in unresectable or metastatic pancreatic cancer patients, either given as a single agent or in combination with cyclophosphamide. Several clinical trials (Table 1) and ongoing clinical trials (Table 2) using CAR T cell mesothelin-targeted immunotherapies in various solid tumors.

**Table 1:** Summary of clinical trials using CAR T cell mesothelin-targeted immunotherapies in various solid tumor

Target	Intervention	Phase	Treated Cancers	Primary outcome measures	Clinical Trials Identifier	Status
Mesothelin	CAR T cells alone	I	Metastatic pancreatic ductal adenocarcinoma (PDAC)	Number of adverse events	NCT01897415	Completed (Well-tolerated and showed preliminary evidence of antitumor efficacy in pancreatic cancer)
	CAR T cells + fludarabine, cyclophosphamide & aldeslekin	I/II	Metastatic cancers (PDAC, cervical, lung, ovarian & mesothelioma)	Frequency and severity of adverse events & objective response rate (ORR)	NCT01583686	Recruiting
	CAR T cells with/without cyclophosphamide	I	Unresectable/ Metastatic PDAC	Number of participants with adverse events	NCT03323944	Active, not recruiting
	CAR T cells with/without cyclophosphamide	I	Metastatic PDAC, epithelial ovarian cancer, & mesothelioma	Number of adverse events	NCT02159716	Completed (No acute adverse events were observed)
CEA	Anti-CEA CAR-T cells	Ib	Liver metastases	Number of participants with adverse events	NCT02850536	Active, not recruiting
		II	Metastatic colorectal cancer	Number of participants with adverse events	NCT02959151	Recruiting
		I	Lung cancer Colorectal cancer Gastric cancer Breast cancer PDAC	Toxicity profile and number of participants with adverse events	NCT02349724	Recruiting
		I	PDAC	Number of patients with tumor response	NCT03267173	Recruiting
MUC-1	Anti-MUC-1 CAR T cells	I/II	Hepatocellular carcinoma Non-small cell lung cancer PDAC Triple-negative invasive breast cancer	Adverse events associated with the administration of anti-MUC-1 CAR T cells	NCT02587689	Recruiting
		I/II	Malignant glioma of brain Colorectal cancer Gastric cancer	Adverse events associated with the administration of anti-MUC-1 CAR T cells	NCT02617134	Recruiting
	Anti-CTLA-4/PD-1 expressing MUC1-CAR-T cells	I/II	Advanced solid tumor	Safety profile of CTLA-4 and PD-1 antibodies expressing MUC1-targeted CAR-T cells	NCT03179007	Recruiting
FAP	Adoptive transfer of re-directed FAP-specific T cells	I	Malignant pleural mesothelioma	Safety profile	NCT01722149	Active, not recruiting

**Table 2:** Summary of ongoing clinical trials using new generation of CAR T cells in solid tumors

Intervention	Phase	Treated cancers	Primary outcome measures	Clinical Trials Identifier	Status
CTLA-4/PD-1 antibody expressing mesothelin-CAR T cells	I/II	Advanced solid tumor	Number of adverse events	NCT03182803	Recruiting
PD-1 antibody expressing mesothelin-specific CAR T cells	I/II	Advanced solid tumor	Safety profile of treatments	NCT03030001	Recruiting
CTLA-4/PD-1 antibody expressing MUC-1 CAR T cells	I/II	Advanced solid tumor	Safety and toxicity profile of treatments	NCT03179007	Recruiting

Targeting of other tumor antigens such as CEA (carcinoembryonic antigen), FAP, and mucin (MUC)-1 also has marked activity in murine models of pancreatic cancer. CEA is a glycoprotein that is highly expressed on the surface of the majority of pancreatic cancer cells. Targeting CEA by CAR T cells has been shown to induce tumor regression and produce long-term tumor eradication in 67% of pancreatic tumor-bearing mice without autoimmune effects. Interestingly, engineered T cells with a dual-receptor CAR (dCAR-T) exert high cytotoxicity against pancreatic tumor cells expressing tumor antigens, CEA and mesothelin, resulting in 80% apoptosis of tumor cell [96]. Pancreatic tumor-bearing mice receiving dCAR-T cells released higher levels of cytokines, including IL-2, IL-6, IFN-λ and TNF-α, and showed a marked reduction in tumor growth compared to controls receiving CAR-T cells alone [96]. However, a CEA-targeted CAR T cell clinical study was halted early, after several treated patients suffered from shortness of breath, highlighting the risks and caution needed when pursuing these studies [122]. Several clinical trials are ongoing to better understand the safety, adverse events and potential effectiveness of CEA-targeted CAR T cells in liver metastases (NCT02850536), colorectal cancer (NCT02959151), and CEA positive cancer, including gastric cancer, lung cancer, pancreatic cancer, breast cancer and colorectal cancer (NCT02349724).

MUC-1 has recently become an interesting target in cancer immunotherapy and it is overexpressed in nearly 90% of pancreatic cancers [123]. Overexpression of MUC-1 has been observed to play a role in tumor progression, invasion, metastasis and therapy resistance. MUC-1 may protect tumor cells from immune surveillance by inhibiting antigen recognition by T cells, thereby, inhibiting the effector function of T cells and promoting an anti-inflammatory TME [124]. Generation of CAR T cells targeting MUC-1 and tested in a MUC-1-expressing breast cancer xenograft mouse model showed that MUC-1-CAR-T cells significantly reduced tumor growth compared to untreated control mice by promoting T cell proliferation and increasing production of inflammatory cytokines such as IFN-γ, resulting in enhanced antitumor immunity and the killing of MUC-1 positive tumor cells [125]. An early phase I study evaluating the therapeutic efficacy and safety profile of CAR T cells targeting MUC-1 in seminal vesicle cancer, revealed no adverse effects. This study showed increased levels of CD4^+^ and CD8^+^ T cells and significant tumor necrosis in treated patients, suggesting that CAR-T cell therapy could be a promising treatment strategy for patients with solid tumors [126]. Clinical trials are ongoing to assess the efficacy and safety of MUC-1-targeted CAR-T cell therapy in patients with relapsed or refractory solid tumors, including pancreatic adenocarcinoma (NCT02587689 & NCT02617134).

New generations of CAR are under investigation to further enhance their activities and specificities, while also decreasing off-target toxicity. It should be noted that the immunosuppressive environment, as well as the effect of upregulation of immune checkpoint inhibitors on CAR T cells, can significantly restrict the full potential of CAR T cell therapy [127, 128]. Therefore, the new generations of CAR constructs incorporate the ability of antigen-redirected T cells to produce immuno-modulatory cytokines such as IL-12 or immune checkpoint inhibitors; this approach enhances infiltration of immune effector cells via the expression of chemokine receptors, and converts immunosuppressive signals into antitumor immune response within the TME [129]. We believe that the efficacy of CAR T cell therapy can be enhanced by combining it with the other chemotherapeutic agents such as cyclophosphamide or immune checkpoint inhibitors such as CTLA-4 and anti-PD-1 mAb [130]. Strategies that combine CAR T cell therapy with immune checkpoint blockade have been studied in murine models. The combination of CAR T cells with PD-1 blockade significantly enhanced tumor regression in comparison with either treatment alone in transgenic mice with lung cancer [131]. Surprisingly, mice treated with combination therapy also showed decreased levels of MDSCs at the tumor site compared with non-treated control mice. Importantly, administration of the combination treatment was well tolerated, with no signs of autoimmunity [131]. Another study demonstrated that the killing activity of CAR T cells was significantly restored upon the addition of anti-PD-L1, suggesting that the efficacy of CAR T cells can be markedly enhanced by blocking PD-L1 immunosuppression. Furthermore, the engineering of CAR T cells to produce immune checkpoint antibodies within the host was shown to be more effective compared with CAR T cells alone, or CAR T cells combined with anti–PD-1 antibody treatment, resulting in enhanced antitumor immunity of CAR T cells and prolonged overall survival of xenograft mouse models [132]. The expression of PD-1 was significantly reduced in anti-PD-1-secreting CAR T cells compared with CAR T cells. Compared with both CAR T cell monotherapy alone and combination therapy, mice treated with anti-PD-1-secreting CAR T cells had a significantly higher ratio of CD8^+^ versus CD4^+^ T cells at the tumor site [133]. Similar results were observed in a renal cell carcinoma mouse model, where CAR T cells secreting anti-PD-L1 antibodies were shown to greatly diminish the exhaustion of T cells and further enhance tumor regression compared with CAR T cells alone [134]. Altogether, these data indicated that this combinatorial strategy could enhance effector function of T cells in the presence of immune checkpoint inhibitors, resulting in tumor regression and improved survival [128]. Ongoing novel clinical studies (NCT03182803 and NCT03030001) are designed to evaluate the efficacy and safety of CAR T cells engineered to express immune checkpoint antibodies in advanced recurrent or refractory malignant solid tumors. Another clinical trial (NCT03179007) is evaluating the safety and efficacy of a novel combination of autologous MUC-1-CAR T cells which express immune checkpoint antibodies in patients with MUC1 positive, advanced solid tumors. Although CAR T cell therapy could produce large populations of T cells specific to tumor antigens, it is time-consuming and expensive compared to vaccine-based therapy [116].

### Targeting the immunosuppressive environment

Targeting immunosuppressive environment enriched with immunosuppressive cells such as TAMs and MDSCs, is an important strategy to the success of immunotherapy in pancreatic cancer. Preclinical studies have elucidated the critical role of TAMs not only in tumor progression and metastasis but also in conferring resistance to chemotherapy and radiotherapy [135]. Furthermore, a higher number of tumor infiltrating immunosuppressive cells always correlate with local or metastatic relapse, leading to reduced survival in pancreatic cancer patients [136]. Thus, Table 3 summarizes intervention strategies using CSF1R blockers, JAK/STAT inhibitors, BTK inhibitors and radiotherapy with or without other checkpoint inhibitors, to target the immunosuppressive environment in tumors.

1. Colony-stimulating factor 1 receptor (CSF1R)

**Table 3:** Intervention strategy with or without other checkpoint inhibitor

Class	Intervention Strategy	Results	Reference(s)
CSF1R	IMC-CS4 (CSF1R mAb) + anti-PD-1 or anti-CTLA-4	↑CTL infiltration ↓ Treg infiltration ↑ Antitumor immunity ↓ Tumor growth	[52, 53]
	PLX-3397 (anti-CSF1R) + durvalumab (anti-PD-L1 Ab)	Ongoing Phase I study in advanced pancreatic and colorectal cancers	NCT02777710
JAK/STAT	Ruxolitinib (JAK/STAT inhibitor)	↓ Tumor growth ↓ PD-L1 expression ↑ CD8+ T cells	[55]
	Ruxolitinib + capecitabine	↑ survival in metastatic pancreatic cancer who failed to response gemcitabine	[58]
BTK	Ibrutinib (BTK inhibitor)	↓ Infiltration of mast cells ↓ Stromal fibrosis ↓ Tumor progression ↓ IL-2 inducible T-cell kinase	[62, 63]
	Ibrutinib + gemcitabine + nab-paclitaxel	Ongoing Phase I/II study in metastatic pancreatic cancer	NCT02562898
	Ibrutinib + gemcitabine + nab-paclitaxel	Ongoing Phase II/III study in metastatic pancreatic cancer	NCT02436668
Radio-therapy (RT)	RT (12 Gy or 5 × 3 Gy) + PD-L1 blocker	↓ Tumor growth ↓ Treg/MDSC infiltration	[64, 65]
	RT + anti-CTLA-4 mAb / anti-PD-L1	Ongoing phase Ib in unresctable, non-metastatic pancreatic cancer	NCT02868632
	RT + ipilimumab (anti-CTLA-4 mAb) / nivolumab (anti-PD-L1)	Ongoing phase II in metastatic pancreatic cancer	NCT02866383
	RT + tremelimumab / MEDI4736	Ongoing phase I/II in unresectable metastatic pancreatic cancer	NCT02311361

In the TME, CSF1R is expressed on TAMs and MDSCs, which play an important role in suppressing cytotoxic immunity. Upon binding to its ligands, CSF-1 or IL-34, CSF1R undergoes oligomerization and autophosphorylation, leading to the activation of signal transduction, and consequently promoting the proliferation, differentiation and survival of macrophages [137]. CSF-1/CSF1R acts a key regulator of the differentiation, recruitment and survival of TAMs. Importantly, TAMs were shown to promote tumor proliferation, angiogenesis, invasion, and metastasis, as well as resistance to therapies [138]. Infiltration of TAMs was shown to be associated with poor survival in cancer patients [139, 140], as a consequence of the overexpression of CSF-1 and immunosuppressive cytokines such as IL-4 and IL-10 in the TME [141].

CSF1R inhibition is associated with reduced immune suppression, enhanced tumor regression and activation of antitumor immune cells as a consequence of a reduced percentage of TAMs to support antigen presentation and T cell activation within the TME [136]. CSF1R was shown to be involved in the recruitment of macrophages in murine models of pancreatic cancer [142]. The administration of the CSF1R inhibitor (AZD7507) caused a reduction in tumor burden and was associated with improved overall survival in murine models. Pro-tumor cytokines such as IL-6 and IL10 levels were reduced in the tumors of mice treated with AZD7507 [142]. Selective depletion of TAMs via inhibition of CSF1R activity in a mouse model resulted in increased CTL infiltration, decreased Treg infiltration [136] and significantly improved efficacy of chemotherapy-induced antitumor immunity, leading to the hypothesis that targeting the CSF1R/CSF1 interaction in combination with immune checkpoint blockade could produce a synergistic response [135]. Zhu and coworkers [136] demonstrated that the efficacy of anti-PD-1 or anti-CTLA4-based immunotherapy was enhanced via CSF1R/CSF1 blockade. It is important to highlight that the tumor growth reduced more than 90% when CSF1R blockade was combined with either anti-CTLA-4 or anti-PD-1 compared to the mice treated only with anti-CTLA-4 or anti-PD-1 alone. Building on these results, a phase I clinical trial (NCT03153410) using the combination of IMC-CS4 (CSF1R mAb) with GVAX and anti-PD-1 is ongoing for patients with borderline resectable pancreatic cancer. Another anti-CSF1R agent, PLX-3397 (pexidartinib) in combination with anti-PD-L1 Ab (durvalumab) is currently in phase I clinical trial (NCT02777710) for patients with advanced pancreatic and colorectal cancers.

(2) Janus Kinase (JAK) inhibitors

The JAK/STAT signaling pathways are essential for a wide range of cytokines and growth factors, leading to critical cellular events such as hematopoiesis and the development of the immune system [143]. Both type I (IFN-α and IFN-β) and type II (IFN-γ) IFNs are potent activators of the JAK/STAT pathway and play a crucial role in cancer immune surveillance and tumor suppression by regulating the expression of PD-L1 through that pathway. It is observed that tumors grow significantly faster in IFN-γ knockout mice than in wild-type animals. However, the level of IFN-γ is higher in pancreatic tumor tissue compared to normal pancreatic tissue [133, 144]. This means that sustained IFN-γ-STAT1 signaling could lead to chronic inflammation and inflammation-mediated tumor development.

Therefore, the over-activation of JAK/STAT signaling is often associated with inflammatory diseases and malignancies [145], the production of inflammatory cytokines and angiogenic factors, and expansion of MDSCs which promote an immunosuppressive TME [146]. Moreover, the JAK/STAT pathway was found to upregulate PD-L1 expression in pancreatic cancer via the activity of IFNs. In an in vivo study of pancreatic tumor cells, flow cytometric analysis revealed that IFNs can upregulate PD-L1 expression, while the expression of PD-L1 induced by IFNs can be diminished by ruxolitinib, a JAK/STAT inhibitor. The administration of ruxolitinib to pancreatic tumor-bearing mice also resulted in significantly reduced tumor growth [147]. Long-term treatment with ruxolitinib is known to decrease the levels of STAT1 and STAT3 phosphorylation, reverse dysregulated development of Th1 and T follicular helper cells (Tfh), and enhance Th17 responses [148]. The inhibition of STAT1 phosphorylation represses upregulation of PD-L1 by IFN-γ, whereas the inhibition of STAT3 phosphorylation decreases the production of immunosuppressive cytokines by tumor cells, resulting in the conversion of tumor-mediated immune suppression to activation of T cells, as well as increased infiltration of CD8^+^ T cells and expression of T-bet, IL-21, perforin, and FasL [147]. In addition, the activation of IL-21 by ruxolitinib is known to act as a costimulatory signal that enhances the effector function of immune cells and activation of T cells, thereby suppressing tumor growth. Consequently, ruxolitinib may be effective in overcoming pancreatic cancer resistance to immune checkpoint therapy. The combination of ruxolitinib with anti-PD-1 exhibited significantly greater efficacy in reducing tumor growth compared with ruxolitinib or PD-1 blockade monotherapy. Additionally, the levels of IFN-γ, CD8^+^ T cells and FasL within TME were significantly higher in tumor-bearing mice treated with combined therapy [144]. All these data indicate that ruxolitinib is effective in facilitating the infiltration and activation of CTLs, thus enhancing the efficacy of immune checkpoint therapy in pancreatic cancer.

In a randomized double-blind, phase II study, ruxolitinib plus capecitabine improved survival in patients with metastatic pancreatic cancer who failed to respond to gemcitabine chemotherapy [149]. Unfortunately, subsequent phase III studies (NCT02117479 & NCT02119663) have closed to enrolment as there was no significant improvement in patient survival. The efficacy of ruxolitinib therapy could be enhanced by using it as an adjunct agent to suppress chronic inflammation and facilitate infiltration of CD8^+^ T cells, rather than as a monotherapeutic agent for overcoming anti-PD-L1 immunotherapy resistance in pancreatic cancer patients [144].

(3) Bruton’s Tyrosine Kinase (BTK) inhibitors

BTK is a member of Tec tyrosine kinase family which is important for B cell development, differentiation and signaling. Activation of BTK has been implicated in the pathogenesis of B cell malignancies as it can trigger downstream signaling events such as proliferation and differentiation mediated through transcription factors such as NFκB, as well as survival signaling cascades such as RAS/RAF/MEK/ERK and PI3K/AKT/mTOR [150]. BTK also leads to T cell suppression by regulating interactions between B cells and macrophages, promoting pancreatic cancer progression [151]. In pancreatic cancer, the infiltration of mast cells was found to correlate with high tumor grade and poor survival [152].

Ibrutinib, a BTK inhibitor, was shown to exhibit anti-fibrotic effects in PDAC by effectively inhibiting infiltration of mast cells in both transgenic mice and patient-derived xenograft models. Ibrutinib reduces stromal fibrosis and inhibits tumor progression, indicating that it may have the potential to sensitize tumors to checkpoint blockade. The administration of ibrutinib in a mouse model of insulinoma, resulted in vascular collapse and tumor regression [153]. Ibrutinib is also known to inhibit interleukin-2-inducible T-cell kinase (ITK), a type of enzyme which is essential for the survival of Th2 cells. As a result, it may shift from Th2 cell protumor response to Th1 cell antitumor response, and augment the deposition of CD8^+^ T cells in tumors [154].

Surprisingly, the combination therapies with ibrutinib and immune checkpoint blockade showed impressive therapeutic effects not only in mouse models of lymphoma that are resistant to ibrutinib, but also in animal models of breast and colon cancers [154]. The depletion of CD4^+^ and CD8^+^ T cells abrogated the treatment efficacy of anti-PD-L1, confirming the role of T cells in activating antitumor activity. Conversely, the antitumor T cell response could be enhanced via the addition of ibrutinib to anti-PD-L1, resulting in tumor regression and prolonged survival of mice with lymphoma [154]. Treatment with ibrutinib or anti-PD-L1 monotherapy exhibited neither delayed tumor growth nor enhanced survival of tumor-bearing mice. In contrast, the combination of ibrutinib and anti-PD-L1 delayed tumor growth, improved survival, and reduced lung metastasis in both breast tumor-bearing mice and colon tumor-bearing mice [154]. Additionally, mice cured by the combination therapies also displayed long term immune memory, as they were resistant to the respective tumors upon tumor re-challenge [154]. A phase I/II clinical trial (NCT02403271) was conducted to assess the safety and efficacy of ibrutinib in combination with anti-PD-L1 antibody (durvalumab) in patients with relapsed or refractory solid tumors, including pancreatic cancer. Another phase II clinical trial (NCT02940301) is recruiting patients with Hodgkin lymphoma to determine the efficacy of ibrutinib in combination with an anti-PD-1 antibody (nivolumab).

(4) Immune checkpoint therapy with radiotherapy (RT)

RT can convert the TME from a ‘cold’ state that lacks infiltration of antitumor immune cells and is resistant to immunotherapy to a ‘hot’ state, which can activate the immune system in triggering an antitumor response, leading to cytotoxicity and the release of stimulatory agents that could enhance the recruitment of T cells to the tumor site. For instance, antitumor T cells that are generated spontaneously or via vaccination may be prevented from entry into the tumor site due to the presence of the desmoplastic stroma of cancer [155]. In contrast, by inducing tumor cell death coupled with the release of danger signals, radiation can stimulate activation and migration of DCs to the tumor-draining lymph nodes where the activation of antitumor T cells will be stimulated. The activated T cells then migrate to the established tumor, kill tumor cells and secrete cytokines, which further enhance activation of DCs and conversion of TAMs to the antitumor M1 phenotype; this indicates that RT converts cold tumor to hot tumors [155].

RT has emerged as a front-runner strategy, where previous studies using murine models of breast cancer showed that it can convert tumors to become responsive to immune checkpoint therapy [156, 157] Interestingly, Azad and coworkers [158] reported that PD-L1 was upregulated after RT and chemotherapy in a JAK/STAT dependent manner, while the intratumoral milieu was shifted away from infiltration of immunosuppressive MDSCs and Tregs towards the infiltration of activated CD8^+^ cells. It is important to note that the tumor response was significantly improved in the Pan02 murine model receiving only higher RT doses (12 Gy or 5 × 3 Gy) plus PD-L1 blockade, whereas anti-PD-L1 alone did not affect tumor growth. PD-L1 blockade did, however, sensitize pancreatic allografts to high RT doses. Flow cytometric analysis revealed that there was a significant increase in infiltration of both CD45^+^CD4^+^ T cells and CD45^+^CD8^+^ T cells upon tumor irradiation, which was further enhanced by blockade of PD-L1. As a result, the authors concluded that the efficacy of RT in delaying tumor growth can be augmented via the blockade of PD-L1, and that the infiltration of MDSCs and Tregs into the tumor site can be significantly decreased by treatment with higher RT doses in combination with PD-L1 blockade. PD-L1 blockade also showed potential in enhancing the anti-metastatic effect of RT in murine liver cancer models, supporting the use of this combination strategy in future clinical studies [158]. These findings were consistent with those of Deng and coworkers [159], who revealed that RT plus anti-PD-L1 could synergistically reduce the infiltration of MDSCs which normally suppress the antitumor T cells response, thus altering the immune response in the TME of breast cancer-bearing mouse models. Although the molecular mechanisms have yet to be fully elucidated, the central message generated by Azad and coworkers [158] clearly provides an important insight on the potential of immune checkpoint inhibitors to radio-sensitize a large group of RT-resistant tumors, including pancreatic cancer that traditionally has been classified as non-immunogenic.

Clinical research on combination strategies is rapidly progressing. There are numerous clinical studies that examine concurrent treatment, in combination with RT, with either PD-L1 blockade or anti-PD-L1 mAbs in various types of solid tumors. Interestingly, unresectable, non-metastatic pancreatic cancer is also being investigated in a phase Ib clinical study (NCT02868632) to evaluate the efficacy of RT plus either anti-CTLA-4 mAb alone, anti-PD-L1 mAb alone, or the combination of both immune checkpoint inhibitors. Recently, a randomized phase II study (NCT02866383) in metastatic pancreatic cancer patients who are intolerant to chemotherapeutic agents is ongoing to determine the efficacy and safety profile of nivolumab or nivolumab plus ipilimumab administered simultaneously with high dose RT; this is estimated to end in 2019. Another similar pilot study (NCT02311361) is also investigating the efficacy of immune checkpoint inhibition (tremelimumab and/or MEDI4736) with RT in unresectable metastatic pancreatic cancer patients.

### Targeting the desmoplastic barrier

Pancreatic cancers are highly desmoplastic, with low vascular perfusion leading to hypoxia, and impeded delivery and efficacy of drugs. The desmoplastic reaction is known to be driven by the production of hyaluronan (HA) by fibroblasts. The accumulation of HA in the TME results in an increase in tumor interstitial fluid pressure (IFP), which significantly compresses blood vessels and impedes blood flow [160]. This in turn leads to the hypoxic environment in the tumor. In the hypoxic microenvironment, tumor cells can achieve tumor escape by upregulating the activation of immunosuppressive cells such as Tregs and MDSCs, which may further lead to the dysfunction of infiltrating CD8^+^ T cells, and consequently, the facilitation of tumor invasion, and metastasis, as well as resistance to therapy [161]. Furthermore, the secretion of immunosuppressive cytokines by the hypoxic TME can induce apoptosis of CD8^+^ T cells and the production of Tregs, along with inhibition of the activation of APCs; this, in turn, results in the failure of DCs to present tumor antigens to T cells for the activation of an antitumor response [161].

Therefore, the treatment strategies which target tumor hypoxia and excessive fibrosis are likely to shift the TME from being immunosuppressive to one that facilitates the activation of T cell immune response and sensitizes pancreatic cancer to immune checkpoint therapy. Focal adhesion kinase (FAK) represents one of the stromal targets and plays a role in cancer cell proliferation, progression and survival [162]. It has been recognized as one of the key factors in regulating the fibrotic TME of PDAC. The overexpression of FAK in many solid tumors is inversely associated with survival [163, 164]. Inhibition of FAK expression with a FAK inhibitor (PF-562,271) in mouse models of pancreatic cancer decreased tumor proliferation and reduced tumor fibrosis; it also reduced the recruitment of MDSCs, Tregs and TAMs into the tumor site. The reduction of myeloid cells was significantly associated with increased levels of CD8^+^ T cells [162, 165]. FAK inhibition also dramatically reduced the secretion of both pro-inflammatory and pro-fibrotic cytokines, such as IL-1α, IL-1β, E-selectin, MMP3, and CCL6 which play a role in the recruitment of myeloid cells [165]. These data suggested that the expression of FAK in pancreatic cancer could facilitate the creation of a fibrotic and immunosuppressive TME that protects tumor cells from CTL-mediated antitumor activity. Additionally, increased infiltration of CD8^+^ T cells, reduced tumor burden and improved overall survival were observed in tumor-bearing mice treated with combination therapy comprising FAK inhibition and PD-1 blockade, compared to PD-1 blockade alone; this suggests that the efficacy of anti-PD-1 can be enhanced by inhibiting the expression of FAK [165]. More conclusive evidence will be available when the phase I/II clinical trial (NCT02758587) is completed. This trial involves patients with advanced solid tumors, including pancreatic cancers, who receive a FAK inhibitor (defactinib) in combination with an anti-PD-1 mAb (pembrolizumab).

Another potential therapeutic target within the stroma of pancreatic cancer is hyaluronan (HA). Accumulation of HA or HA combined with its binding proteins (HABPs) forms a size selective barrier to antitumor immune cells and the efficient delivery of therapeutic drugs; this creates an immunosuppressive environment which prevents infiltration of T cells, as well as preventing chemotherapeutic agents and mAbs from entering the tumor and reaching their site of action. Its accumulation is thus always associated with poor prognosis in cancer patients [160]. A novel investigational agent, PEGPH20 (pegvorhyaluronidase alfa), which is a PEGylated form of recombinant human hyaluronidase, PH20, has been identified as an enzyme that can degrade HA [160, 166]. PEGPH20-induced HA depletion resulted in increased recruitment of NK cells and antibody delivery into the high HA tumor site, greatly enhancing cetuximab or trastuzumab ADCC [167]. The increased infiltration of CD8^+^ T cells as a consequence of the degradation of HA by PEGPH20 also improved the efficacy of anti-PD-L1 in HA-rich breast cancer tumor models, resulting in inhibition of tumor growth, in comparison with PEGPH20 or anti-PD-L1 alone [168]. The combination of PEGPH20 and anti-PD-L1 also rendered HA-rich tumors sensitive to anti-PD-L1. Imaging studies showed that there was an accumulation of anti-PD-L1 within the TME which was thought to be enhanced by PEGPH20 [168]. These data suggested that removal of HA by PEGPH20 enhanced the efficacy of immune checkpoint therapy by increasing infiltration of CD4^+^ T cells and NK cells, while decreasing the percentage of MDSCs; at the same time, it also enhanced the accumulation of immunotherapeutic antibody in HA-rich tumors.

PEGPH20 has also been shown to delay tumor growth and metastasis and to enhance chemotherapy efficacy in HA-rich tumor models, including pancreatic cancer. This is likely due to the enhanced perfusion of therapeutic agents into the tumor. In the PEGPH20-treated KPC mouse model, the diameter of vessels was significantly increased, resulting in improved blood flow. Surprisingly, when PEGPH20 was administered together with gemcitabine, the combination regimen significantly suppressed tumor growth and increased apoptosis resulting in an increased overall survival compared to gemcitabine alone [166, 169]. Moreover, a preclinical studydemonstrated that in mice treated with a combination of PEGPH20 and shIDO-ST, a *Salmonella*-based therapy which targets the immunosuppressive molecule indoleamine 2,3-dioxygenase (IDO), there was a significant reduction in tumor burden, an increased infiltration of immune cells into the desmoplastic stroma of pancreatic cancer, and an enhancement of FasL-mediated apoptosis of tumor cells [170]. These results suggested that stromal barriers to infiltration of antitumor immune cells can be overcome with PEGPH20, with a consequent enhancement of the antitumor activity of immunotherapy. However, the actual mechanism of HA depletion induced by PEGPH20 is still under investigation [171].

To date, there are no clinical studies investigating PEGPH20 as monotherapy or combinatory treatment in cancers [172]. Numerous clinical trials are currently enrolling patients; these trials will evaluate the efficacy of PEGPH20 in combination with other therapeutic agents (Table 4). A non-randomized pilot study (NCT02921022) investigating the effects of PEGPH20 plus a combination regimen (gemcitabine, nab-paclitaxel and rivaroxaban) in patients with/without prior thromboembolic events is currently ongoing. A Phase I clinical trial (NCT03481920) is currently underway to evaluate the pharmacodynamics and efficacy of PEGPH20 plus avelumab (anti-PD-L1 mAb) in chemotherapy-resistant pancreatic cancer patients [173]. Another clinical trial (NCT03267940) is ongoing to evaluate the safety, tolerability and antitumor activity of PEGPH20 with anti-PD-L1 in patients with cholangiocarcinoma/gallbladder cancer. The safety and tolerability of a combination of PEGPH20 and anti-PD-1 is also under investigation in HA-high patients with lung cancer and gastric cancer (NCT02563548). Targeting the tumor stroma could modulate its immunosuppressive conditions by facilitating the normalization of blood vessels and weakening the immunosuppressive effects of cancer-associated fibroblasts; this will effectively enhance the transportation of oxygen and therapeutic agents as well as the infiltration of effector T cells to the tumor tissues, thereby, enhancing the efficacy of the immune checkpoint therapies [174].

**Table 4:** Summary of ongoing studies to evaluate safety profile and efficacy of PEGPH20 in multiple cancers

Intervention	Phase	Targeting Cancers	Primary Outcome Measures	Clinical Trial Identifier
PEGPH20 + pembrolizumab	II	Metastatic pancreatic cancer	Progression free survival (PFS)	NCT03634332
PEGPH20 + gemcitabine + RT	II	Localized, unresectable pancreatic cancer	Number of participants with adverse events	NCT02910882
PEGPH20 + gemcitabine + nab-paclitaxel + rivaroxaban	N/A	Advanced pancreatic cancer	Rate of symptomatic thromboembolic events	NCT02921022
PEGPH20 + gemcitabine + nab paclitaxel vs Placebo + gemcitabine + nab-paclitaxel	III	Stage IV previously untreated pancreatic cancer	PFS & Overall Survival	NCT02715804
PEGPH20 + gemcitabine + nab-paclitaxel vs PEGPH20 alone	II	pancreatic cancer	Pathologic complete response, clinically relevant pancreatic fistula	NCT02487277
PEGPH20 + avelumab	I	Chemotherapy resistant pancreatic cancer	Overall response rate & safety profile	NCT03481920
PEGPH20 + pembrolizumab	Ib	Relapsed/ refractory non-small cell lung cancer & gastric cancer	Overall response rate	NCT02563548
PEGPH20 + eribulin mesylate vs eribulin mesylate alone	Ib/II	Metastatic breast cancer	Recommended phase II dose, overall response rate	NCT02753595
PEGPH20 + Cisplatin (CIS) + Gemcitabine (GEM) vs PEGPH20 + Atezolizumab + CIS + GEM vs CIS + GEM	I	Cholangiocarcinoma & Gallbladder cancer	Treatment adverse events and objective response rate	NCT03267940

### Combinations of mismatch repair deficiency and PD-1 blockade in tumors

Surprisingly, the effectiveness of immunotherapy can be predicted based on the presence of mismatch repair deficiency. Mismatch repair deficient (dMMR) cancers are hypothesized to have large numbers of mutation-associated neoantigens (MANAs) which can be recognized by the host antitumor immune cells [175, 176]. dMMR is associated with frameshift mutations that take place within the coding sequences, resulting in the production of functionally inactive proteins that can be presented via MHC molecules to CD8^+^ T cells as tumor neoantigens, consequently eliciting an antitumor immune response as well as the infiltration of T cells into the TME [177]. An early clinical trial (NCT02060188) showed that colorectal cancer with mismatch repair deficiency was associated with an increased tumor neoantigen load and infiltration of immune effector cells, consequently enhancing tumor sensitivity to immune checkpoint blockade, particularly anti-PD-1 [178]. A phase II clinical trial (NCT01876511) is ongoing to evaluate the clinical activity of pembrolizumab (anti-PD-1) in patients with dMMR tumors. The objective response rate (ORR) and progrssion-free survival were significantly higher (40 and 78% respectively) in dMMR cancer patients compared with MMR-proficient colorectal cancer patients ((0 and 11%, respectively) [179], supporting the hypothesis that dMMR tumors are more responsive to immune checkpoint blockade than MMR-proficient tumors.

### Future prospects

Pancreatic cancer has been recognized as one of the most aggressive malignancies and is usually diagnosed at an advanced level, with limited or no effective therapeutic options thus far. However, preclinical and clinical trials have shown promising results in therapies targeting immune checkpoint molecules. Of note, immune checkpoint therapies is largely ineffective in pancreatic cancer due to the low mutational load along with the hypoxic pancreatic TME that is filled with immunosuppressive cells, which acts as a selective barrier to drug penetration and infiltration of immune effector cells, significantly limiting the efficacy of immunotherapy. Many efforts have been made to gain insights into patients who exhibit resistance towards immune checkpoint therapy and to find ways to maximize treatment efficacy via combination therapies. Rational combinations of immunotherapy may represent one potential strategy to synergistically overcome the immunosuppressive microenvironment of pancreatic cancer, as well as to induce long-lasting antitumor activity within the TME. The optimal dose, schedule, and ideal sequence, for example, when to combine immunotherapy with other therapies such as chemotherapy, radiation therapy, or targeted agents, must be determined, as these therapies have different mode of actions [180]. In view of the increasing number of novel compounds being synthesized [181, 182] or isolated from natural products [183–190], more pre-clinical studies are required to test whether these new classes of compounds can also target the tumor microenvironment of pancreatic cancer. These new compounds may act synergistically with chemotherapy, radiation therapy, immunotherapy or other targeted therapies.

Additionally, several studies are being conducted to develop (1) vaccine-based therapy with immune checkpoint blockade to increase infiltration of T cells; (2) co-delivery of stromal targeting agents with immune checkpoint inhibitor; and (3) co-delivery of T cells priming agents with immunotherapeutic antibodies [191]. Interestingly, the current focus has shifted to characterization of tumor neoantigens. In pancreatic cancer, assessment of the quality of tumor neoantigens has opened a new avenue for investigating tumor progression. Advancement in neoantigen discovery may unlock the potential of personalized cancer vaccines to work alone or in combination with other therapies to enhance the strength of antitumor effects and improve clinical outcomes. Future efforts can be anticipated to harness neoantigen-specific antitumor immunity to treat patients with immune checkpoint inhibitor-resistant cancers such as pancreatic cancer and to identify immunogenic hotspots for directed neoantigen targeting [192]. Personalized immunotherapy based on individual genetic information, molecular biology and immune profiling is hypothesized to result in the greatest clinical outcomes for cancer patients [193], leading either to the conversion of untreatable cancer into a controllable chronic disease or long term tumor eradication [194]. In addition, more efforts should be made in the development of databases and bioinformatics platforms, as an enormous amount of genomics and biomarkers data have been generated; at the same time, this could empower the practice of precision immune-oncology. More validation studies and well-designed clinical trials are warranted to provide evidence to support personalized immunotherapy for patients with pancreatic cancer.

## Conclusion

The development of combination strategies that act to stimulate the immune response and break down the barriers of TME in conjunction with immunotherapies, hold promises for improving the overall survival of pancreatic cancer patients. To achieve this, researchers and clinicians need to advance their understandings of tumor immunology, identify novel biomarkers, optimize the timing of immunotherapy, and implement novel preclinical models to better predict the therapeutic efficacy; there also needs to be an improvement in clinical trial designs to achieve a better understanding of the mechanism of action and resistance of pancreatic cancer towards immunotherapy. We believe that combination immunotherapies represent a promising modality in pancreatic cancer; these are advancing from bench to bedside at a rapid pace. Translation of immunotherapies into clinical practice will bring a new hope for patients suffering from this aggressive, silent, asymptomatic killer.

## Abbreviations

ACT: Adoptive T cell transfer
APC: Antigen presenting cells
Arg: Arginine
Arg-1: Arginase-1
BTK: Bruton’s Tyrosine Kinase
CAR: Chimeric antigen receptor
Cox-2: Cyclooxygenase-2
CSF1R: Colony-stimulating factor 1 receptor
CT-011: Pidilizumab
CTLA-4: Cyototoxic T-lymphocyte antigen-4
DC: Dendritic cells
ECM: Extracellular matrix
FAP: Fibroblast activation protein
FDA: Food and Drug Administration
GM-CSF: Granulocyte macrophage-colony stimulating factor
HA: Hyaluronan
HABP: Hyaluronan binding proteins
IFN: Interferon
IFP: Interstitial fluid pressure
iNOS: Inducible nitric oxide synthase
ITK: Inducible T-cell kinase
JAK: Janus kinase
mAb: Monoclonal antibody
Mb: Megabase
MDSC: Myeloid-derived suppressor cells
MIP-3α: Macrophage inflammatory protein-3 alpha
MMPs: Matrix metalloproteinases
MUC-1: Mucin-1
NK: Natural killer
NSCLC: Non-small cell lung cancer
ORR: Objective response rate
OS: Overall survival
PD-1: Programmed death 1
PDAC: Pancreatic ductal adenocarcinoma
PD-L1: Programmed death-ligand1
PEGPH20: Pegvorhyaluronidase alfa
PFS: Progression free survival
PNT: Peroxynitrite
ROS: Reactive oxygen species
RT: Radiotherapy
SD: Stable disease
TAA: Tumor associated antigen
TAM: Tumor associated macrophages
TCR: T cell receptors
TGF-α: Transforming growth factor-α
TGF-β: Transforming growth factor-β
TIL: Tumor infiltrating lymphocytes
TME: Tumor microenvironment
TNF-α: Tumor necrosis factor-α
TP: Thymidine phosphorylase
Treg: Regulatory T-cells
VEGF: Vascular endothelial growth factor
